# Chloroform

**Published:** 1848-09

**Authors:** 


*Chloroform*—By R. D. Mussey, M. D., Professor of Surgery at Cincinnati, Ohio.

Dear Sir,—Some time since, I sent you a short communication, expressing a favorable opinion of chloroform as an anæsthetic agent in surgical operations. I now write to say, that a more extended experience with this article has served to confirm the good opinion of it I then entertained.

I have performed *fifty-nine* operations upon patients nnder its influence, and there has not been a single instance in which it has left unpleasant effects. I still prefer it to ether, on account of the smallness of the quantity required, the greater certainty of its making the requisite impression, the greater expeditiousness of its operation, the less irritation it gives to the glottis, its inferior liability to agitate the voluntary muscles, and the more speedy evanescence of its influence after the patient has ceased to inhale it.

In all cases the operations have been attended with *very littte* or *no pain;* often an entire suspension of consciousness; sometimes a full consciousness of what was going on, without the sensation of pain. Within the present week, I amputated the thigh of a much-valued clergyman for an extensive ulceration of the knee-joint. He was much reduced in flesh and strength, and his nervous sensibility was so great that he contemplated the operation with the deepest horror. He was put under the influence of the chloroform while the limb was removed and the wound dressed, When he awoke and found that the operation was over, he clasped his hands and gave thanks to God for having bestowed a boon of such priceless value upon the suffering family of man.

Yesterday I operated upon a teamster for strangulated hernia. The strangulation had existed forty-eight hours; the tenderness of the abdomen, the persevering vomiting of fæcal matter, a small and frequent pulse, great restlessness, and prostration consequent upon free bloodletting, the tobacco glyster, and the other means which had been employed for relief before my arrival, offered an uncertain prospect for ultimate success. The chloroform was given, and its influence kept up through the operation. The man appeared to have got into difficulty with his *horses,* spoke roughly to them, and talked incoherently during the whole time occupied in the operation. After he had roused up a little, I asked him if we had hurt him? he said "no, except a little when you tried to pull me off the potatoe cart." This morning he was better; slept a good deal through the night, and considerable hope is entertained of his recovery.

In two cases of lithotomy, I have operated while the patients were kept still with the chloroform. Neither of them felt pain. One, a boy of 8 years, the other a boy of 12. The latter, sensitive and obstinate, was brought and confined upon the operating table only by force. When everything was in readiness for giving the chloroform, he requested that I would *not begin to cut till he was asleep* After he had made a few inspirations, observing his eyelids drooping, I asked him if he were sleepy; he said, *yes, but don't cut till I am asleep.* In a few moments his eyes were closed, and the muscles of his limbs were soft. I then proceeded to the operation, extracted the stone, and was about to untie him, when he opened his eyes, and said, "*doctor, don't begin to cut till I get to sleep.*"

When administered to patients in the horizontal position with an empty stomach, and introduced gradually into the lungs, the safety of this agent need not be questioned. There is a dose of arsenic or opium that can kill a patient, and at the same time another dose that is entirely safe; it is so, I believe, with chloroform; and I cannot help regarding it as one of the most important gifts for the relief of human suffering, that a kind Providence has sent us in this or any other age.—*Boston Med. and Sur. Jour.*

Cincinnati, July 5th. 1848.

